# Association between histological diaphragm atrophy and ultrasound diaphragm expiratory thickness in ventilated patients

**DOI:** 10.1186/s40560-022-00632-5

**Published:** 2022-08-19

**Authors:** Irene Dot, Purificación Pérez-Terán, Albert Francés, Yolanda Díaz, Clara Vilà-Vilardell, Anna Salazar-Degracia, Roberto Chalela, Esther Barreiro, Alberto Rodriguez-Fuster, Joan Ramon Masclans, Judith Marin-Corral

**Affiliations:** 1 Critical Care Department, Hospital del Mar, Barcelona, Spain; 2 Critical Illness Research Group (GREPAC), Institut Hospital del Mar d’Investigacions Mèdiques (IMIM), Barcelona, Spain; 3 Urology Department, Hospital del Mar, Barcelona, Spain; 4 Respiratory Medicine Department-Muscle Wasting and Cachexia in Chronic Respiratory Diseases and Lung Cancer Research Group, Institut Hospital del Mar d’investigacions mèdiques (IMIM), Barcelona, Spain; 5 Centro de Investigación en Red de Enfermedades Respiratorias (CIBERES), Instituto de Salud Carlos III (ISCIII), Barcelona, Spain; 6 Health and Experimental Sciences Department (CEXS), Universitat Pompeu Fabra, Barcelona, Spain; 7 Thoracic Surgery Department, Hospital del Mar, Barcelona, Spain; 8 Division of Pulmonary Diseases & Critical Care Medicine, University of Texas Health San Antonio, Sant Antonio, TX USA; 9 Respiratory Department, Hospital del Mar, Barcelona, Spain

**Keywords:** Diaphragm thickness, Critical illness-associated diaphragm weakness, Atrophy, Muscle, Dysfunction, Diaphragm ultrasound

## Abstract

**Background:**

Diaphragm fiber atrophy has been evidenced after short periods of mechanical ventilation (MV) and related to critical illness-associated diaphragm weakness. Atrophy is described as a decrease in diaphragm fiber cross-sectional area (CSA) in human diaphragm biopsy, but human samples are still difficult to obtain in clinics. In recent years, ultrasound has become a useful tool in intensive care to evaluate diaphragm anatomy. The present study aimed to evaluate the ability of diaphragm expiratory thickness (Tdi) measured by ultrasound to predict diaphragm atrophy, defined by a decrease in diaphragm fiber CSA obtained through diaphragm biopsy (the gold standard technique) in ventilated patients.

**Methods:**

Diaphragm biopsies and diaphragm ultrasound were performed in ventilated donors and in control subjects. Demographic variables, comorbidities, severity on admission, treatment, laboratory test results and evolution variables were evaluated. Immunohistochemical analysis to determine CSA and ultrasound measurements of Tdi at end-expiration were performed, and median values of the control group were used as thresholds to determine agreement between them in further analysis. Sensitivity, specificity, and positive and negative predictive values of an ultrasound Tdi cutoff for detecting histologic atrophy were calculated. Agreement between two ultrasound observers was also assessed.

**Results:**

Thirty-five ventilated organ donors and 5 ventilated controls were included, without differences in basic characteristics. CSA and Tdi were lower in donors than in controls. All donors presented lower CSA, but only 74% lower Tdi regarding control group thresholds. The cut-off value for lower diaphragm expiratory thickness (Tdi < 1.7 mm) presented a sensitivity of 73%, a specificity of 67%, a positive predictive value of 96% and a negative predictive value of 17% for determining the presence of diaphragm atrophy (CSA < 2851 μm^2^).

**Conclusions:**

Diaphragm atrophy and thickness reduction is associated to MV. While a lower Tdi in diaphragm ultrasound is a good tool for diagnosing atrophy, normal or increased Tdi cannot rule atrophy out showing that both parameters should not be considered as synonymous.

## Background

Diaphragm fiber atrophy after the use of mechanical ventilation (MV) has been described as an important factor contributing to the development of critical illness-associated diaphragm weakness [1, 2]. Its prevalence in the intensive care unit (ICU) in ventilated patients is 40–80% according to previous studies and has been associated with a high rate of weaning failure, increased length of stay, and increased ICU and hospital mortality [3–8]. For these reasons, its detection, diagnosis, and evaluation are crucial to improve the management of critically ill patients.

Animal and human studies have revealed the presence of histological atrophy (i.e., reduction in muscle fiber size) after a few hours of controlled MV or inappropriate ventilator settings with a decreased force-generating capacity [9–13]. MV itself seems to affect the diaphragm, but many other factors such as inflammation, malnutrition and the use of certain pharmacological agents contribute to this problem in the ICU. Although muscle biopsy and histological evaluation through fiber cross-sectional area (CSA) is still the best methodology to evaluate muscle structure, it is an invasive procedure that may cause severe complications in routine use and is rarely performed in clinical practice [14].

In recent years, ultrasound has become increasingly popular in the day-to-day management of ICU patients given its simplicity, its noninvasive nature, and its high safety profile. It can be used to assess distinctive diaphragmatic characteristics such as the diaphragm expiratory thickness or Tdi and offers high reproducibility [15, 16]. Rapid early changes in Tdi have been described after MV and its decrease in ultrasound studies has been accepted as an indicator of a loss of diaphragm muscle mass [17, 18]. Although it has been widely used to describe the atrophy found in the diaphragm of critically ill patients who have undergone MV, non-association between variation in Tdi and muscle fiber atrophy has been demonstrated in a recent animal model by Aarab et al. [19]. Unfortunately, this issue has not been examined in human studies.

We hypothesized that Tdi would be a good tool for diagnosing diaphragm atrophy in ventilated patients. The aims of this study were: (1) to assess the ability of Tdi measured at end-expiration by ultrasound to evaluate diaphragm fiber size and atrophy (assessed by diaphragm CSA) in ventilated patients; and (2) to analyze the impact of MV on diaphragm CSA and Tdi. We also aimed to give a scientific and experimental basis for the use of lower muscle thickness measured by ultrasound to define atrophy.

## Methods

### Study subjects

Critically ill organ donor patients admitted in a University hospital ICU during a period of 3 years and who required MV for a period longer than 24 h, were eligible for the study. The donor group comprised two types of donors, donors after circulatory death (those included in the Maastricht III classification), in whom circulatory death occurred after a controlled and planned withdrawal of life-sustaining therapies, and brain-dead donors, diagnosed as such in view of the evidence of a lack of cortical and brainstem reflexes secondary to irreversible brain damage. We also included a control group comprising patients who had undergone elective thoracic surgery for localized lung nodule. Exclusion criteria were as follows: age younger than 18, previous diaphragmatic surgery and difficulty obtaining diaphragm ultrasound images. Protocols were approved by the Ethics Committees of Hospital del Mar (2017/7183/I) and informed consent was obtained from all subjects or their next of kin prior to inclusion. The procedures used in this study adhered to the tenets of the Declaration of Helsinki. Further details regarding study donors are detailed in a previous study by Marin-Corral et al. [11].

### Clinical data

At inclusion, demographic variables such as age, gender, body mass index (BMI), toxic habits, chronic medical comorbidities, chronic medications, and type of admission were recorded for all subjects. In the donor group the severity scores in the first 24 h after admission (Acute Physiology and Chronic Health Evaluation II or APACHE II and Sequential Organ Failure Assessment or SOFA) were also recorded, as were the type of donation, treatments received during ICU stay (systemic corticosteroids, type and days of sedation, use and number of days with neuromuscular blockers, insulin, enteral nutrition, and vasoactive drugs), ventilator settings and laboratory test results on the day of inclusion. Finally, ICU complications and evolution (days on MV, ICU length of stay) were also described.

### Biopsies and fiber cross-sectional area (CSA)

**Fig. 1:**
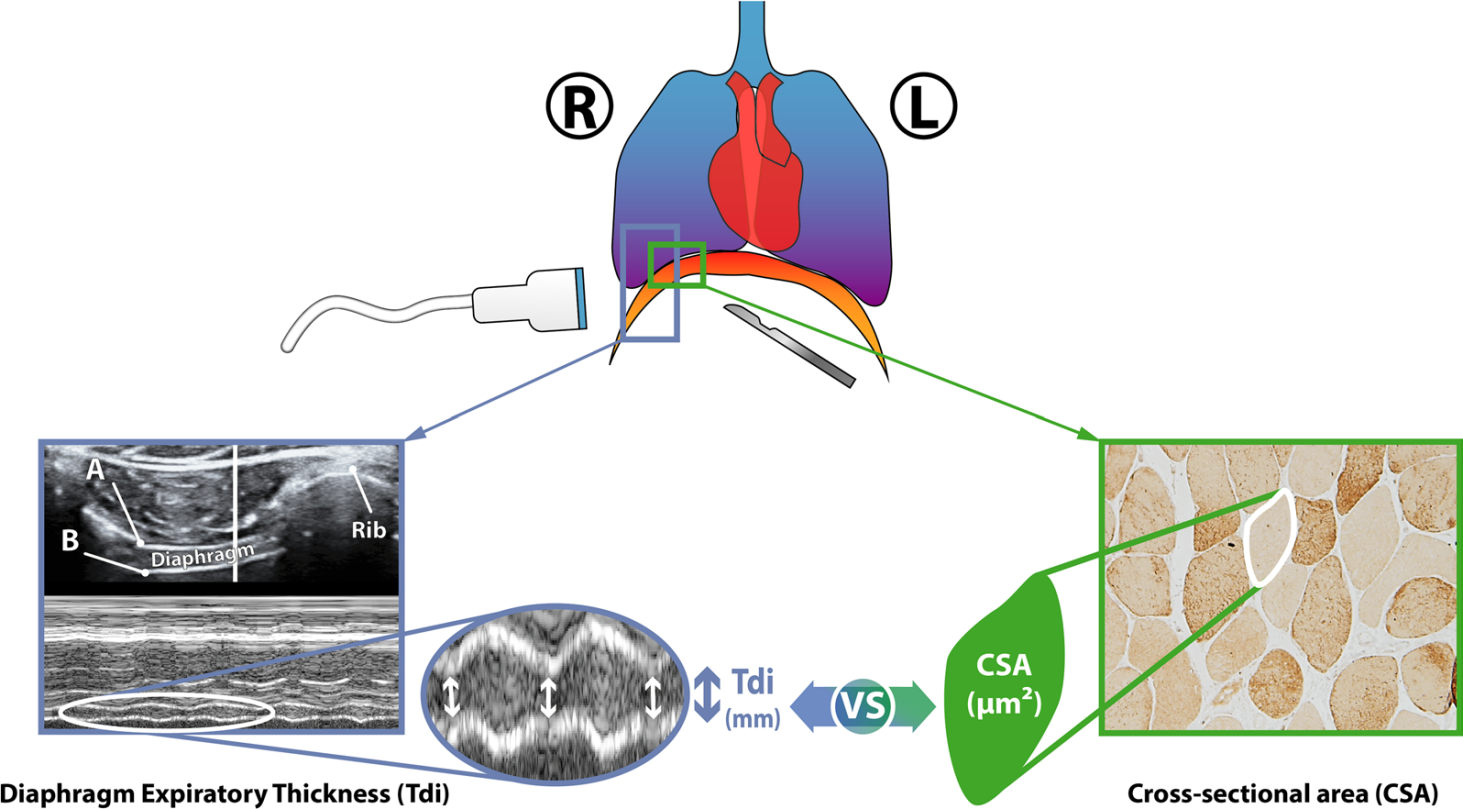
Representative image of the methodology followed for the study: location of the sample collection, performance of the diaphragmatic ultrasound and measurements of the CSA and Tdi

In all patients included, diaphragm biopsy samples were obtained early during surgery, from the anterior right costal diaphragm, lateral to the insertion of the phrenic nerve (Fig. 1). Specifically, in the case of donors, the biopsy was obtained as soon as possible during organ removal, ensuring minimal ischemia time due to hypoperfusion. Muscle samples were immediately immersed in an alcohol–formol bath and then embedded in paraffin. These tissues were used for the histological analysis. Morphometric analyses were carried out in the diaphragm samples. CSA and proportions of myofibers expressing myosin heavy chain I (MHCI or type I, Sigma-Aldrich, Saint Louis, MO, USA) or II (MHCII or type II, Sigma-Aldrich, Saint Louis, MO, USA) were assessed by immunohistochemical analysis under light microscopy (Olympus, Series BX61, Olympus Optical Co., Hamburg, Germany) coupled with an image-digiting camera (Olympus, Series DP71, Olympus Optical Co., Hamburg, Germany) and evaluated using a specific morphometry program (Image J, U. S. National Institutes of Health, Bethesda, Maryland, USA). At least 150 fibers in both type I and type II staining were measured and counted in each muscle specimen. Average value of all measured CSA values was calculated for all included patients. Further details on biopsy procedures are detailed elsewhere [11].

### Diaphragm expiratory thickness ultrasound (Tdi)

Diaphragm ultrasound (Vivid I^®^ ultrasound device, General Electric, Fairfield, CT, USA) was performed in the six hours before organ donation (donor group) or prior to surgery (control group) with subjects connected to MV in a semi-recumbent position (45º). Tdi (mm) was measured at end-expiration in the zone of apposition of the right hemidiaphragm to the rib cage with the linear probe (10–12 MHz) located in the 9th–10th intercostal space, at the level of the anterior axillary line in a transversal position as previously described. In this position, the diaphragm is visualized as a three-layered structure which thickens with inspiration. The two external echogenic layers correspond to the pleura and the peritoneum. Between the two layers, diaphragm muscle is clearly visualized (Fig. 1). To measure Tdi at end-expiration, M-mode was used to obtain three uninterrupted and well-defined respiratory cycles. Images were stored in digital format and Tdi was measured from the inner-to-inner layer with an electronic caliper. The average of the three individual values were used. Measurements were analyzed independently by two intensivists experienced in ultrasound, and intraobserver/interobserver agreement was evaluated.

### Statistics

Continuous variables were described as means and standard deviation (SD) or medians and interquartile range [IQR (25–75)] according to whether the Kolmogorov–Smirnov test showed the distribution to be normal or non-normal. Categorical variables were described as number of cases and percentages [*n* (%)]. Chi-square, Fisher’s test, Student’s *t*-test, and Mann–Whitney *U* test were used to evaluate variables at baseline. Agreement between the two ultrasound observers was performed with Spearman’s Rho correlation coefficient, and intraindividual variability was assessed by the variation coefficient.

We established cut-off values for histologic atrophy and ultrasound Tdi decrease in our population, based on the characteristics of our control group as previously described (i.e., the median values of CSA and Tdi) [20]. Standard formulas were used to calculate the sensitivity, specificity, positive predictive value and negative predictive value of the ultrasound Tdi cutoff to detect histologic atrophy through CSA. Data were analyzed using the statistical package for social sciences 15.0 BM^®^ SPSS Statistics^®^, Chicago, IL, USA). Statistical significance was established at p ≤ 0.05.

## Results

### Characteristics of patients

Forty mechanically ventilated patients were included in the analysis, 35 organ donors and 5 controls. Table 1 shows the characteristics of the patients included. Briefly, no significant differences in age, gender and BMI were found between the groups. The control group had a higher prevalence of smoking (60% vs 34%, p > 0.05) and of chronic comorbidities (80% vs 43%, p > 0.05) without reaching statistical significance. There were no differences between groups in terms of chronic treatment such as oral corticosteroids, statins or insulin. Most of the donors (91%) were admitted to the ICU for medical causes (neurological illnesses), and as described above all the controls were admitted for elective surgery.

**Table 1:** Basic characteristics of controls and donors

	Controls *n* = 5	Donors *n* = 35	*p*-value
Demographics			
Age, years	61 (56–77)	69 (55–76)	ns
Gender, female	2 (40)	14 (40)	ns
Body mass index, kg/m^2^	25 (3)	26 (5)	ns
Toxic habits			
Smoking	3 (60)	12 (34)	ns
Alcoholism	2 (40)	5 (14)	ns
Comorbidities			
COPD	1 (20)	3 (9)	ns
Diabetes mellitus	0 (0)	7 (20)	ns
Heart failure	0 (0)	3 (9)	ns
Chronic kidney disease	1 (20)	2 (6)	ns
Hematologic disease	1 (20)	0 (0)	ns
Previous treatment			
Oral corticosteroids	0 (0)	0 (0)	ns
Statins	2 (40)	14 (40)	ns
Insulin	0 (0)	2 (3)	ns
Type of ICU admission			
Elective surgery	5 (100)	1 (3)	< 0.001
Medical cause	0 (0)	32 (91)	
Traumatic cause	0 (0)	2 (6)	

Data expressed as frequencies and percentages [*n* (%)] or medians and interquartile ranges (IQR or 25th–75th percentile). *COPD* chronic obstructive pulmonary disease, *ICU* intensive care unit

Clinical characteristics of donors during their ICU stay before inclusion are shown in Table 2. Summarizing, donors had a median APACHE score at admission of 29 (23–33) and a median SOFA score of 7 (5–9). Half were brain-dead donors (54%) and the other half were Maastricht III (45%). A total of 18 (51%) patients presented some complication during the ICU stay, most frequently of infectious nature (34%) without any diagnoses of septic shock. All the patients were ventilated in controlled modalities, with low positive end expiratory pressures (PEEP). ICU length of stay and duration of MV were identical because all patients were admitted intubated and connected to invasive MV for a median of 5 (2–9) days.

**Table 2:** Clinical characteristics of organ donors

	Donors *n* = 35
Severity scores at admission	
APACHE II	29 (23–33)
SOFA	7 (5–9)
Donation type	
Brain-death	19 (54)
Maastricht III	16 (45)
Pharmacological treatments during ICU	
Systemic corticosteroids	12 (34)
Neuromuscular blockers	12 (34)
Days on neuromuscular blockers	1 (1–2)
Benzodiazepines	24 (68)
Days on benzodiazepines	1 (0–3)
Opioids	26 (74)
Days on opioids	2 (0–3)
Insulin	12 (34)
Subcutaneous	31 (88)
Intravenous	3 (8)
Norepinephrine	27 (77)
Maximal dose, µg/kg/min	0.3 (0.2–0.6)
Dobutamine	4 (11)
Maximum dose, µg/kg/min	7 (1–16)
Enteral nutrition	18 (51)
Days on enteral nutrition	2 (0–6)
Enrolled on a physical therapy program	4 (11)
Complications during ICU stay	
Hyperglycemias > 200 mg/dl	17 (48)
Infectious	12 (34)
Non-infectious	6 (17)
Ventilator settings	
Controlled modalities	35 (100)
PEEP, cmH_2_O	7 (6–8)
Analytical variables on day of inclusion	
C-reactive protein, mg/dl	2 (0–17)
Troponin, ng/l	41 (8–485)
Creatine kinase, U/L	179 (54–581)
Albumin g/dl	3 (0.5)
ICU evolution data	
Days on mechanical ventilation	5 (2–9)
ICU length of stay	5 (2–9)

Data expressed as frequencies and percentages [*n* (%)] or medians and interquartile ranges (IQR or 25th–75th percentile). *APACHE II* Acute Physiology and Chronic Health Evaluation II, *SOFA* Sequential Organ Failure Assessment, *ICU* intensive care unit

### Diaphragm cross-sectional area

**Fig. 2:**
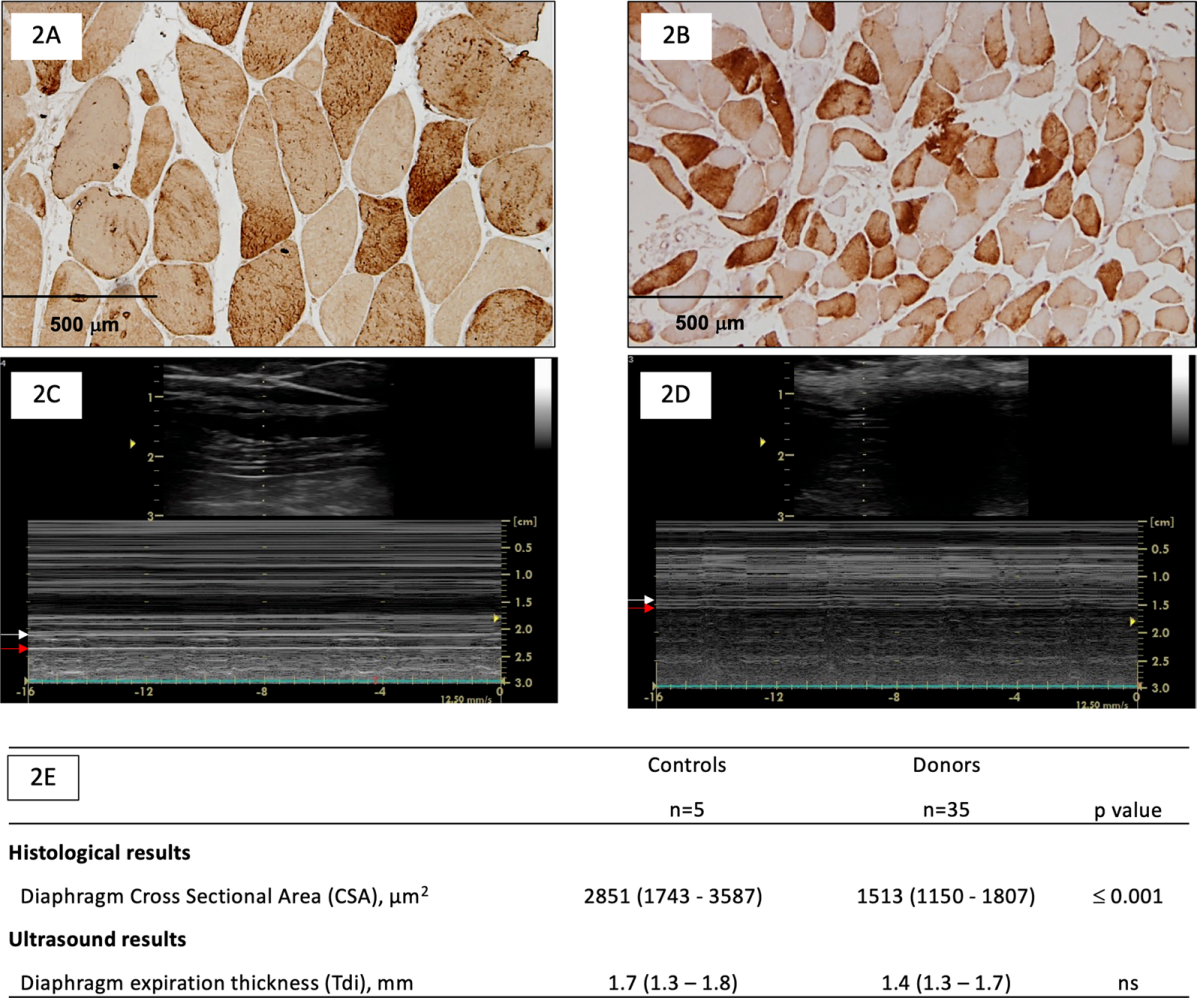
Diaphragm cross-sectional area (CSA) and diaphragm expiratory thickness (Tdi) related data in donor and control groups. **A** and **B** Representative examples of histological samples. **C** and **D** Representative examples of ultrasound measurements. White arrow: pleural layer; red arrow: peritoneal layer. **E** Principal study variables (histological and ultrasound) in control and donor groups. Data are shown as median and interquartile ranges (IQR or 25th–75th percentile)

The size of diaphragmatic fibers was significantly lower in donors than in controls [1513 (1150–1807) μm^2^ vs 2851 (1743–3587) μm^2^, p ≤ 0.001] (Fig. 2A, B and E). The median CSA value in the control group (2851 μm^2^) was used as a threshold to define diaphragm atrophy.

### Diaphragm expiratory thickness (Tdi)

**Fig. 3:**
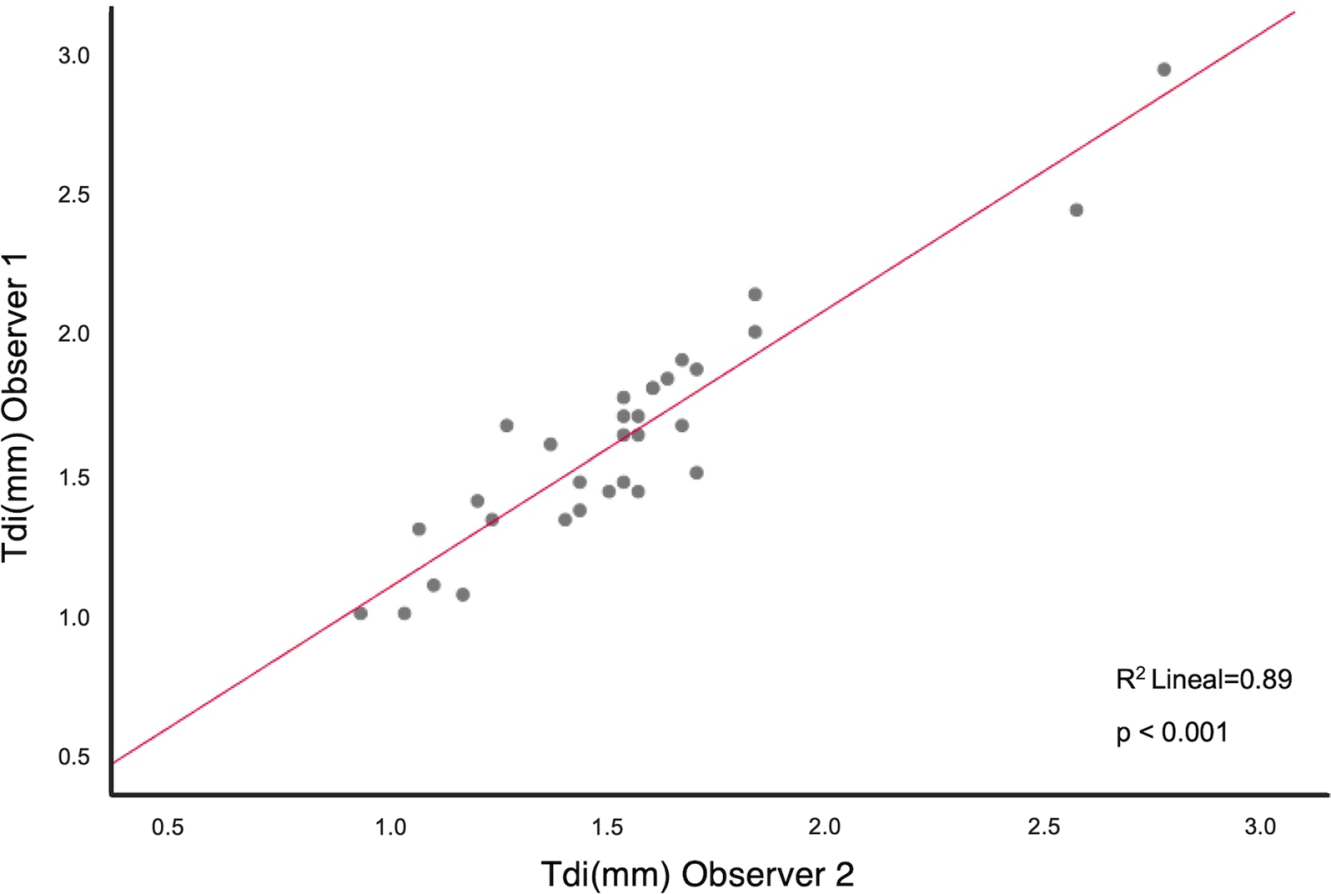
Correlation of diaphragm expiratory thickness (Tdi) measured by diaphragm ultrasound between two observers

Ultrasound Tdi intra-observer coefficient of variation was 0.93 (CI 0.8–0.96) and 0.96 (CI 0.94–0.98) according to sonographer. Measurements of Tdi showed a good inter-observer agreement between the two observers (Rho 0.89, p ≤ 0.001) as shown in Fig. 3. Tdi was lower in donors than in controls although the difference did not reach statistical significance [1.4 (1.3–1.7) mm vs 1.7 (1.3–1.8) mm, p > 0.05] (Fig. 2C, D and E). The median Tdi value in the control group (1.7 mm) was used as a threshold to define lower Tdi at end-expiration.

### Association between histological atrophy and ultrasound expiratory thickness

**Fig. 4:**
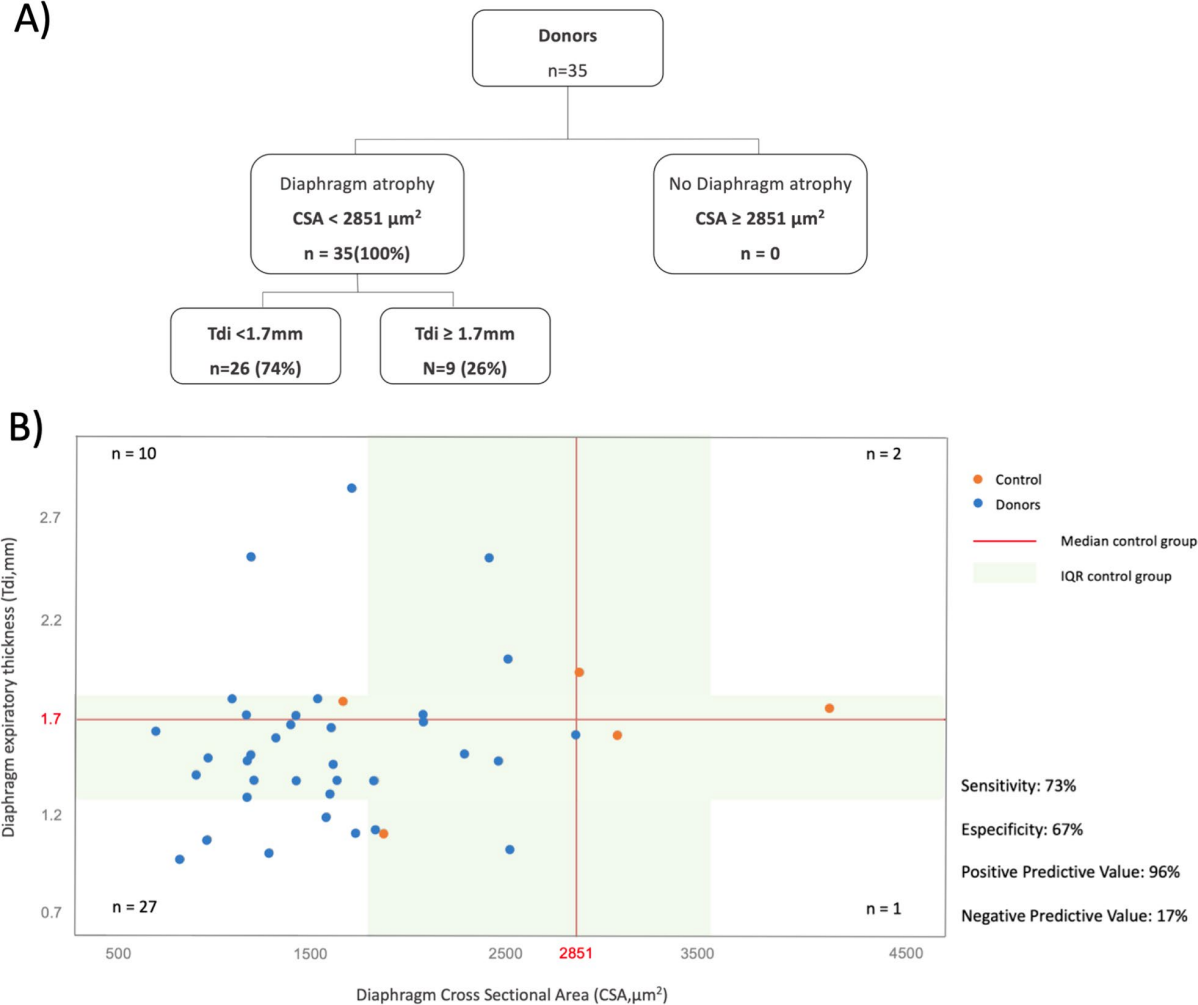
Association between histological atrophy and ultrasound diaphragm expiratory thickness. **A** Donor’s flowchart with diaphragm expiratory thickness measured by diaphragm ultrasound regarding the presence of diaphragm atrophy. **B** Distribution of diaphragm expiratory thickness (Tdi) related to diaphragm cross-sectional area (CSA) in all study population

All ventilated donors included presented histological diaphragm atrophy, while only 74% of them presented lower Tdi at end-expiration (Fig. 4A). Figure 4B shows the distribution of Tdi related to diaphragm CSA in all study population. The cut-off value for lower diaphragm expiratory thickness (Tdi < 1.7 mm) presented a sensitivity of 73% (56–86), a specificity of 67% (10–99), a positive predictive value of 96% (84–99) and a negative predictive value of 17% (7–34) for determining the presence of diaphragm atrophy (Fig. 4B).

## Discussion

The key findings of this study are that (1) the presence of diaphragm atrophy and decreased diaphragm expiratory thickness is associated to MV; (2) diaphragm expiratory thickness measured by ultrasound (Tdi) is able to detect diaphragm atrophy, but does not have the capacity to rule it out completely; and (3) diaphragm ultrasound is a good tool for diagnosing diaphragm atrophy in ventilated patients; it presents good reproducibility and is easy to perform at the bedside.

Our results for diaphragm atrophy in ventilated patients are in agreement with previous studies. A reduction of diaphragm myofiber CSA has been shown after the use of short periods of controlled MV in comparison with control patients [13, 21]. In 2008, Levine et al. were the first to demonstrate this phenomenon in brain-dead adult donors [9]. Since then, the results have been replicated in other groups of ventilated patients with different pathologies [11, 22]. Because each of these studies used its own control group, there are no generalized normality values for diaphragm CSA. In our cohort, as threshold for determining diaphragm atrophy, we considered the median CSA value of the control group, which is similar to the thresholds previously used in the literature [9, 21–25]. As previously described, our results show that all our ventilated donors had histological atrophy according to the control group threshold after a median of 5 days under MV, probably related to the time-dependent relationship between atrophy and time of ventilation observed in previous studies [13].

Given the difficulties involved in obtaining diaphragm biopsies, recent studies have used diaphragm ultrasound as an accessible tool for exploring diaphragm structure in critical patients. Tdi in expiration has been used as a surrogate of muscle mass and has been accepted in ultrasound studies as a noninvasive assessment of diaphragm atrophy in ventilated patients [18, 26]. Changes in Tdi have been detected in mechanically ventilated patients after the first hours on MV [17, 27, 28]. Again, the lower limit values of Tdi range widely (between 1.6 and 2.3 mm) in healthy and ventilated patients in the different studies [17, 19, 29–34]. The cut-off value obtained from our control group and used as a threshold to define a lower Tdi agrees with previous data (1.7 mm). In our cohort difference in Tdi between control and donor groups did not reach the statistical significance probably because of the small sample size.

At present, it is unclear whether a Tdi measured by ultrasound should be accepted as an indicator of histological diaphragm fiber size. In our study, although 100% of the donors had diaphragm atrophy, only 74% presented decreased Tdi. This suggests that both variables probably do not represent the same phenomenon. Reynolds et al., studied the diaphragm of ventilated pigs through histology and diaphragm ultrasound, assessing diaphragm CSA and Tdi in both ventilated non-paced and ventilated animals after the use of phrenic nerve pacing. Although the aim of the study was not to correlate the two measures, ventilated pigs in the non-paced group showed a decrease in both CSA and Tdi in comparison with treated animals, suggesting a possible association between the two [35]. More recently, Aarab et al. provided exhaustive histological, ultrasound and functional information about diaphragm of ventilated piglets showing that a decrease in its stiffness quantified by shear wave ultrasound elastography was associated with a smaller fiber CSA but not with a decrease in diaphragm expiratory thickness [19]. Our results in a human population of ventilated patients agree with these findings confirming that ultrasound diaphragm thickness may increase or decrease according to the combination of changes in all its components including not only the muscle fibers (atrophy or hypertrophy), but also connective tissue, the vessels, inflammatory or lipid accumulation and muscle edema which may also be altered by the conditions that frequently affect critically ill patients [11, 21, 36–38]. In this study we used histological CSA as a gold standard for evaluating atrophy. Our results suggest that Tdi < 1.7 mm is able to detect atrophy in 73% of cases (sensitivity) and Tdi ≥ 1.7 mm to rule it out in 67% (specificity). We also found that when Tdi detects a decrease in thickness, it is well correlated with histological atrophy in 96% of cases (positive predictive value). Notably, when Tdi is within normal or increased ranges, there is no atrophy in only 17% of cases, suggesting the presence of other mechanisms rather than fiber size determining muscle thickness. Then, muscle atrophy evaluated by diaphragm ultrasound could go unnoticed when diaphragm expiratory thickness is increased by other mechanisms including other anatomical structures evaluated by ultrasonography (e.g., edema). Interestingly, DiNino et al. described a minimum Tdi value for extubating success of 1.7 mm [39].

In this study, two independent observers performed the diaphragm ultrasound measurements, with intra and inter-observer agreements on Tdi close to the values previously described in the literature. This confirms that diaphragm ultrasound in ventilated patients is an easy-to-perform and reproducible tool at the bedside [15].

### Study limitations

The current study has important limitations. First, as several previous studies, we included our own control group using its median CSA and Tdi as the reference or normal threshold. The sample size of the control group is small given the difficulties in obtaining diaphragmatic biopsies in healthy subjects but similar to those reported till present. Nevertheless, values on both fibrillar size (CSA) and diaphragm expiratory thickness (Tdi) are also within the ranges previously described in the literature for similar sized control groups. It is necessary to carry out studies with a sufficient sample size to establish normal ranges in these variables, which would probably have to be stratified according to age or gender or BMI. Second, because our aim was limited to evaluating the diaphragm structure at the same timepoint through two different methods, our study did not include data on diaphragmatic function nor diaphragmatic change of thickness between two timepoints, both parameters with important clinical value. Future studies should focus on including these variables to decide on the best cut-off values for clinicians to use in their day-to-day practice. Third, because our objective was focused on the comparison of both techniques, we did not check the mechanisms underlying the absence of lower Tdi in donors with atrophy. Further studies should address this issue by measuring some variables related to connective tissue, inflammation, lipid accumulation or edema. Fourth, biopsies were not obtained guided by ultrasound, but surgery procedure made accessible the costal diaphragm in the apposition zone, were all the ultrasound were performed. Fifth, we only measured the right hemidiaphragm expiratory thickness because, as described elsewhere, the left side tends to be poorly visualized [3]. However, in cases in which the left side can be seen clearly, no differences have been reported between the hemidiaphragms and so the evaluation of just one of them should not introduce a bias [16].

## Conclusions

The presence of a decreased diaphragm CSA and Tdi measured by diaphragm ultrasound is associated to MV. Diaphragm ultrasound seems to be a good tool for diagnosing atrophy, but the terms CSA and Tdi do not represent the same muscle structural anatomy and should not be used synonymously. Because that normal or increased Tdi cannot rule out diaphragm atrophy, and consequently protective diaphragm MV strategies should be used in all ventilated patients, not only in those with lower Tdi. Future studies should seek to clarify the association between these structural findings and functional variables to avoid diaphragm myotrauma and thus improve outcomes.

## Data Availability

The datasets used and/or analyzed during the current study are available from the corresponding author on reasonable request.

## Abbreviations

MV: Mechanical ventilation
ICU: Intensive Care Unit
CSA: Cross-sectional area
Tdi: Diaphragm expiratory thickness
APACHE II: Acute Physiology and Chronic Health Evaluation II
SOFA: Sequential Organ Failure Assessment
PEEP: Positive end expiratory pressure
MHCI: Myosin heavy chain I
MHCII: Myosin heavy chain II
SD: Standard deviation
IQR: Interquartile range
